# Roles of a Novel Crp/Fnr Family Transcription Factor Lmo0753 in Soil Survival, Biofilm Production and Surface Attachment to Fresh Produce of *Listeria monocytogenes*

**DOI:** 10.1371/journal.pone.0075736

**Published:** 2013-09-16

**Authors:** Joelle K. Salazar, Zhuchun Wu, Weixu Yang, Nancy E. Freitag, Mary Lou Tortorello, Hui Wang, Wei Zhang

**Affiliations:** 1 Institute for Food Safety and Health, Illinois Institute of Technology, Bedford Park, Illinois, United States of America; 2 Department of Microbiology and Immunology, University of Illinois College of Medicine, Chicago, Illinois, United States of America; 3 United States Food and Drug Administration, Bedford Park, Illinois, United States of America; 4 Food Safety Research Center, Shanghai Institute for Biological Sciences, Chinese Academy of Sciences, Shanghai, China; 5 School of Biology and Pharmaceutical Engineering, Wuhan Polytechnic University, Wuhan, China; University of Iowa Carver College of Medicine, United States of America

## Abstract

*Listeria monocytogenes* is a foodborne bacterial pathogen and the causative agent of an infectious disease, listeriosis. *L. monocytogenes* is ubiquitous in nature and has the ability to persist in food processing environments for extended periods of time by forming biofilms and resisting industrial sanitization. Human listeriosis outbreaks are commonly linked to contaminated dairy products, ready-to-eat meats, and in recent years, fresh produce such as lettuce and cantaloupes. We identified a putative Crp/Fnr family transcription factor Lmo0753 that is highly specific to human-associated genetic lineages of *L. monocytogenes*. Lmo0753 possesses two conserved functional domains similar to the major virulence regulator PrfA in *L. monocytogenes*. To determine if Lmo0753 is involved in environmental persistence-related mechanisms, we compared *lmo0753* deletion mutants with respective wild type and complementation mutants of two fully sequenced *L. monocytogenes* genetic lineage II strains 10403S and EGDe for the relative ability of growth under different nutrient availability and temperatures, soil survival, biofilm productivity and attachment to select fresh produce surfaces including romaine lettuce leaves and cantaloupe rinds. Our results collectively suggested that Lmo0753 plays an important role in *L. monocytogenes* biofilm production and attachment to fresh produce, which may contribute to the environmental persistence and recent emergence of this pathogen in human listeriosis outbreaks linked to fresh produce.

## Introduction

*Listeria monocytogenes* is a Gram-positive soil saprophyte and causative agent of a human foodborne infectious disease, listeriosis. *L. monocytogenes* infection in healthy adults may result in a spectrum of clinical illnesses ranging from general influenza-like symptoms such as fever, chills, and headache, to gastrointestinal symptoms including vomiting and diarrhea, which usually last one to four days in duration [1]–[3]. However, for immunocompromised individuals, such as infants, the elderly and pregnant women, listeriosis infections typically develop to more severe complications such as meningitis and encephalitis [1], [4], [5] leading to a mortality rate of 20% [1], [6]. The U. S. Centers for Disease Control and Prevention estimates that 1,662 invasive infections of listeriosis occur annually in the U. S., causing 1,520 hospitalizations and 266 deaths [7].

Human listeriosis outbreaks are commonly linked to contaminated dairy products and ready-to-eat (RTE) poultry and meats [4], [8]. However, in recent years, several major listeriosis outbreaks were associated with *L. monocytogenes* contaminated fresh produce in the U.S. For instance, in 2009, twenty cases of listeriosis were linked to contaminated sprouts [7]. In 2010, ten cases of listeriosis and five deaths were linked to chopped celery [9], [10]. In 2011, a large *L. monocytogenes* foodborne outbreak was traced back to contaminated whole cantaloupes, which led to 147 cases of infections from 28 states, 142 cases of hospitalizations (97%) and 33 deaths (22%) including one miscarriage [11]. Two serotypes of *L. monocytogenes*, i.e. 1/2a and 1/2b, were identified in this outbreak [12].

In a previous study [13], we identified a putative Crp/Fnr family transcription factor Lmo0753 which was highly specific to human outbreak-associated genetic lineages of *L. monocytogenes*. Lmo0753 shares two conserved functional domains with the well-known positive regulatory factor A, or PrfA [14]. Crp/Fnr family transcription factors can respond to various environmental stimuli, such as changes in temperature, pH, and nutrient availability, and subsequently trigger the expression of many stress response and virulence-related genes [5]. It has been reported that PrfA is necessary for biofilm formation in *L. monocytogenes* [15], [16] and aids in environmental survival and abiotic surface attachment [17]. Because of the amino acid sequence similarity between Lmo0753 and PrfA, we hypothesize that Lmo0753 may also play a role in environmental persistence-related mechanisms in *L. monocytogenes* such as biofilm formation and surface attachment. To test this hypothesis, we compared the relative ability of *lmo0753* deletion mutants in *L. monocytogenes* str. 10403S and str. EGDe, with their respective wild types and complementation mutants for growth under different nutrient availability and temperatures, survival in soil, biofilm productivity and surface attachment to lettuce leaves and cantaloupe rinds.

## Results

### Growth kinetics under different nutrient availability and temperatures

**Figure 1:**
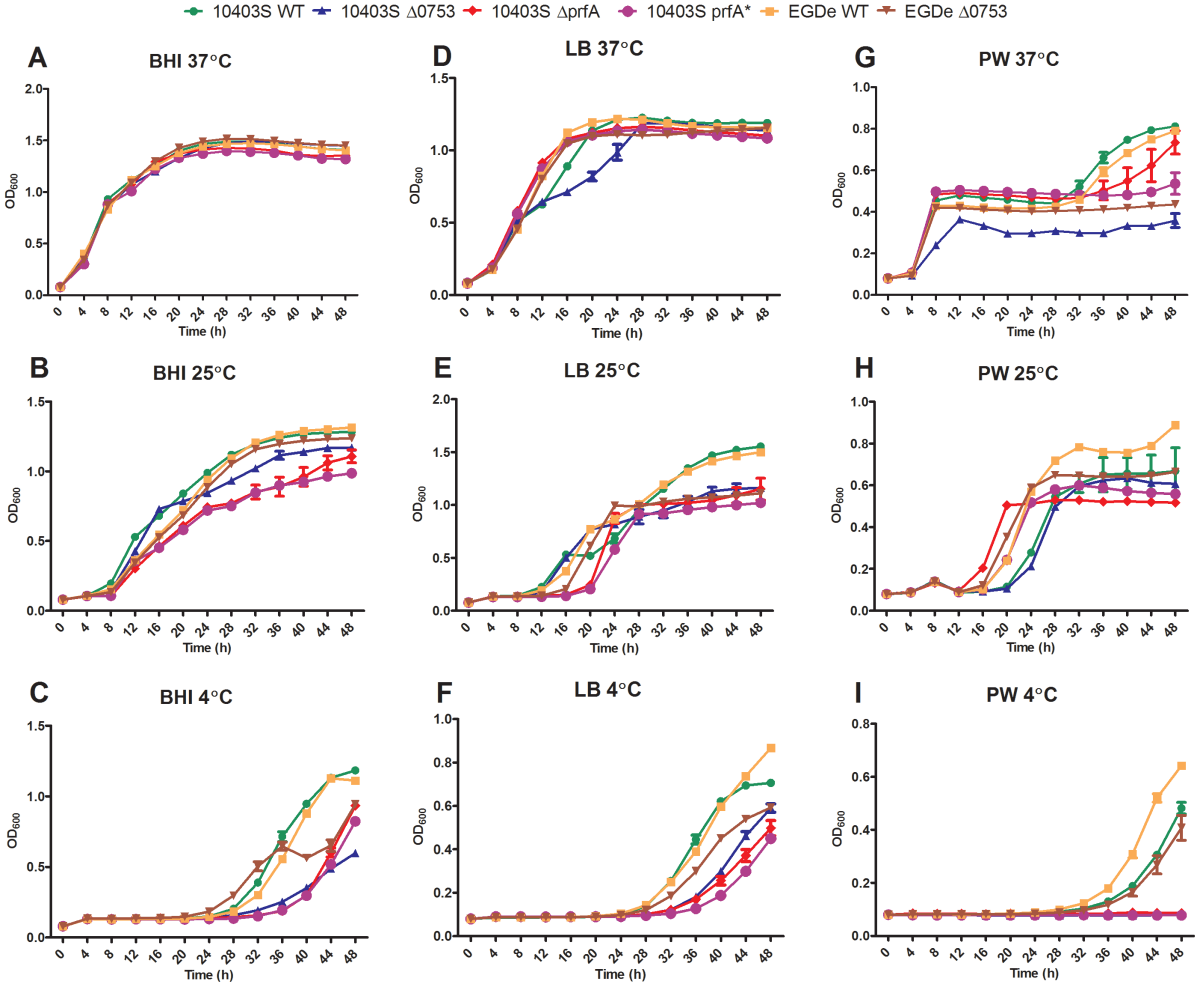
Growth of *L. monocytogenes* strains in broth culture. Standard deviations represent three independent experiments.

A standard curve including linear regression for *L. monocytogenes* 10403S wild-type strain at 37°C is given in Figure S1. When grown at 37°C, no significant difference in growth was evident among mutants and wild-types in BHI (Fig. 1A). 10403S *Δ0753* showed a decreased population in LB compared to its parent strain between 12 h to 24 h (Fig. 1B). After 24 h, the population level of 10403S *Δ0753* increased to that of the parent strain. When grown in PW (Fig. 1C), some differences were observed between the mutants and wild-type strains. For instance, both 10403S and EGDe wild-type strains reached a population of OD_600_ 0.8 after 48 h, whereas their respective *Δ0753* mutants had populations of approximately 0.35 and 0.45, respectively. Both complementation mutants in 10403S and EGDe (JS-c0753) were able to restore the growth phenotype of the wild-types (data not shown). This indicates that deletion of *lmo0753* has an impact on the growth of *L. monocytogenes* at 37°C when environmental nutrients are limited. Similarly, 10403S *ΔprfA* and 10403S prfA* showed decreased maximum populations as compared to their parent strains.

When grown at 25°C, the growth defects of the *Δ0753* mutants were more obvious compared to their parent strains. In BHI, both wild-type strains and EGDe *Δ0753* reached maximum populations of OD_600_ 1.3 after 48 h, whereas the 10403S *Δ0753* mutant only reached a maximum population of OD_600_ 1.1 (Fig. 1D). In LB, both wild-type strains reached maximum populations of OD_600_ 1.4 after 48 h, while their respective *Δ0753* deletions only reached populations of approximately OD_600_ 1.0 (Fig. 1E). Both 10403S *Δ prfA* and 10403S prfA* showed decreased populations similar to the *Δ0753* mutants and increased lag phases when grown in LB medium. In PW, growth differences were observed between parent strains (Fig. 1F): EGDe wild-type reached the maximum population of OD_600_ 0.9 and 10403S wild-type reached a maximum population of OD_600_ 0.65. 10403S *Δ0753* mutant had a population similar to that of its parent strain, however, the EGDe *Δ0753* mutant had a maximum population of OD_600_ 0.65, representing a significant decrease (*p*<0.05). The EGDe *Δ0753* (JS-c0753) complementation strain was able to restore the growth phenotype of the parent strain.

When grown at 4°C, both wild-type strains reached the same maximum population, OD_600_ 1.2, after 48 h in BHI (Fig. 1G). EGDe *Δ0753* reached a maximum population of OD_600_ 0.9, with a decrease in population from 36 to 40 h that was not seen with its parent strain. 10403S *Δ0753* had a greater decrease in maximum population as compared to its parent strain, OD_600_ 0.6. Both 10403S *ΔprfA* and prfA* grew similarly to the 10403S *Δ0753* mutants. In LB, similar growth patterns were observed. EGDe wild-type reached a maximum population of OD_600_ 0.85 after 48 h, whereas its *Δ0753* mutant only reached a maximum population of 0.6 (Fig. 1H). 10403S wild-type reached a maximum population of OD_600_ 0.7 after 48 h, whereas the respective *Δ0753* mutant only reached 0.45. In PW, only the wild-type strains and EGDe *Δ0753* showed growth after 48 h (Fig. 1I). The EGDe parent strain reached a maximum population of OD_600_ 0.7 after 48 h and its *Δ0753* mutant reached a population of approximately 0.4. 10403S and EGDe *Δ0753* (JS-c0753) complementation mutants were able to restore these growth phenotypes to that of the wild-type strains.

### Biofilm production

**Figure 2:**
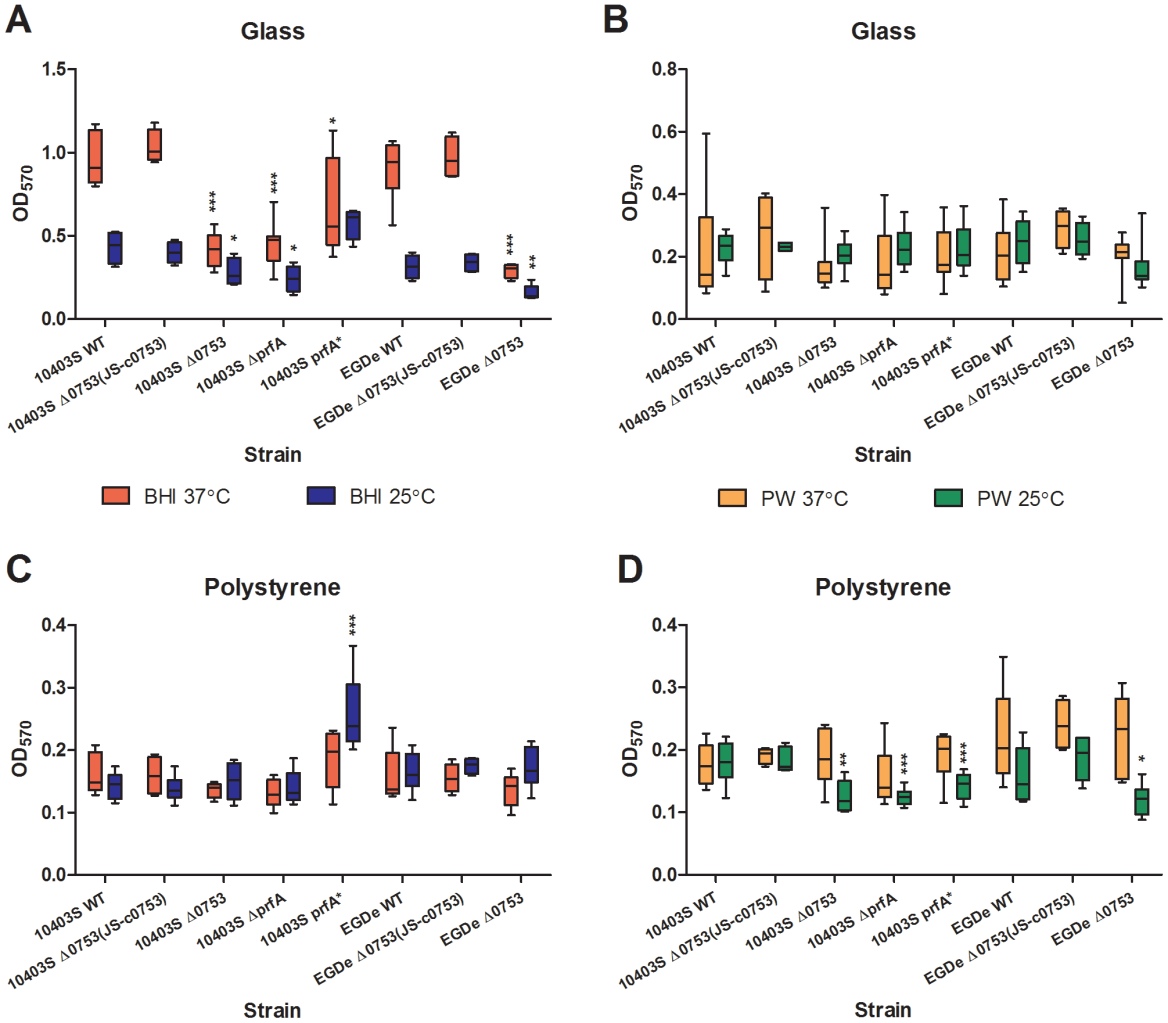
Biofilm production of *L. monocytogenes* strains. The bacterial biofilm formation is quantified by OD_570_ readings. A) Quantification of biofilm formation in glass test tubes. B) Quantification of biofilm formation in polystyrene 96-well plates. Middle horizontal line in each box represents the median of the entire data set; the upper and lower horizontal lines represent the upper quadrant median and the lower quadrant median, respectively. Standard deviations represent three independent experiments. Significant differences in comparison to the parent strains under the same condition are shown as * (*P*<0.5), ** (*P*<0.001), and *** (*P*<0.0001).

As shown in Fig. 2A, when grown on a glass surface in BHI at 37°C, 10403S *Δ0753*, 10403S *ΔprfA*, and EGDe *Δ0753* all displayed significant decrease in biofilm production compared to their parent strains (*P*<0.0001). 10403S prfA* showed a significant increase in biofilm production as compared with its parent strain (*P*<0.05). When grown on a glass surface in BHI at 25°C (Fig. 2A), 10403S *Δ0753*, 10403S *ΔprfA*, and EGDe *Δ0753* also showed less biofilm productivity in comparison with their parent strains (significant at *P*<0.05 for 10403S *Δ0753* and *ΔprfA* strains and at *P*<0.001 for EGDe *Δ0753*). However, no significant difference was observed when the mutants were grown on a glass surface in PW at 37°C or 25°C (Fig. 2B). Both 10403S and EGDe *Δ0753* (JS-c0753) complementation mutants were able to restore the biofilm phenotype to those of the wild-type strains. These results showed that deletion of *lmo0753* led to decreased biofilm production of *L. monocytogenes* on a glass surface when grown in nutritious media.

Some statistical differences in biofilm production were also observed between the mutants and respective wild-type strains when they were grown on a polystyrene surface (Fig. 2C and 2D). For instance, when grown in BHI at 25°C (Fig. 2C), 10403S prfA* showed a significant increase in biofilm production as compared to its parent strain (*P*<0.0001). In PW at 25°C (Fig. 2D), 10403S *Δ0753* (*P*<0.001), 10403S *ΔprfA* and 10403S prfA* (*P*<0.0001), and EGDe *Δ0753* (*P*<0.05) all displayed reduced biofilm production compared with their respective parent strains. Both 10403S and EGDe *Δ0753* (JS-c0753) complementation mutants were able to restore the biofilm productivity to those of the wild-type strains, suggesting that *lmo0753* may be required for biofilm production in *L. monocytogenes* on polystyrene when grown in a less nutritious environment at 25°C.

### *L. monocytogenes* attachment to plant surfaces

**Figure 3:**
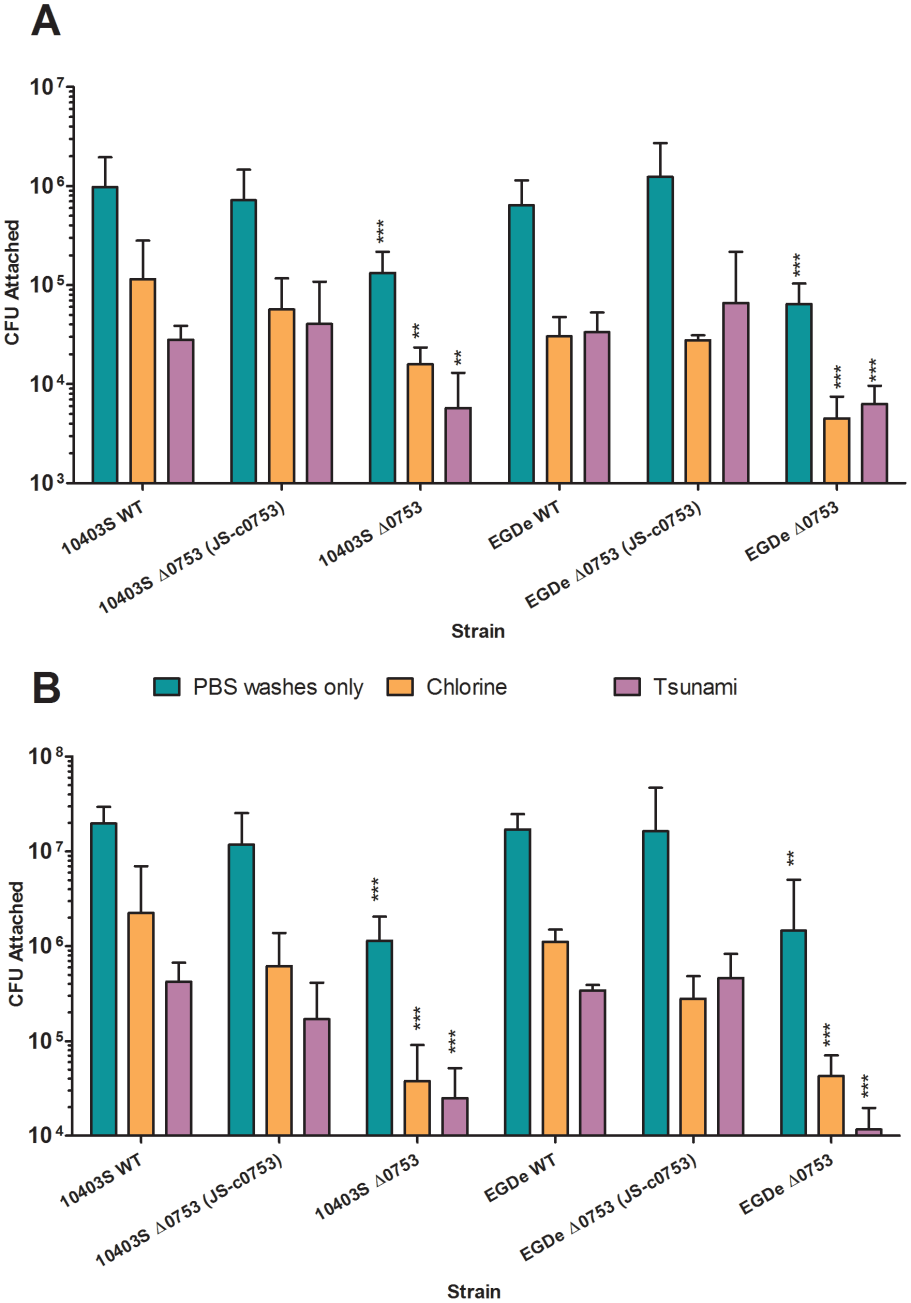
*L. monocytogenes* attachment to fresh produce surface. A) Bacterial attachment to romaine lettuce. B) Bacterial attachment to cantaloupe rind. Standard deviations represent three independent experiments. Significant differences in comparison to the parent strains under the same condition are shown as * (*P*<0.5), ** (*P*<0.001), and *** (*P*<0.0001).

To determine if *lmo0753* is associated with *L. monocytogenes* attachment to food plant surface, we compared the relative ability of the mutants and wild-type strains to attach to romaine lettuce leaves (Fig. 3A) and cantaloupe rinds (Fig. 3B). Compared to the wild-type strains and complementation mutants, 10403S *Δ0753* and EGDe *Δ0753* both showed significantly (P<0.0001) decreased attachment to lettuce leaves and cantaloupe rinds without sanitizer treatment.

To further evaluate if sanitizer treatment would be effective to remove surface-attached *L. monocytogenes*, we gently treated the inoculated lettuce leaves and cantaloupe rinds with sodium hypochlorite (or chlorine) or Tsunami 100 solutions, both of which are commonly used by the fresh produce industry. When surface-attached *L. monocytogenes* on romaine lettuce was treated with chlorine, an approximate 3-log reduction was achieved for 10403S and its complement mutant and an approximate 3.5-log reduction was seen for EGDe and its complement mutant. Under the same treatment, 10403S *Δ0753* and EGDe *Δ0753* mutants showed an approximate 3.5-log (*P*<0.001) and 4-log (*P*<0.0001) reduction, respectively. When treated with Tsunami 100, an approximate 3-log reduction was seen for both 10403S and EGDe and their respective complements; whereas the respective *Δ0753* mutants showed an approximate 4-log reduction (*P*<0.001 for 10403S and *P*<0.0001 for EGDe).

Washing of surface-attached cantaloupe rinds by *L. monocytogenes* with chlorine resulted in an approximate 1.5-log reduction for 10403S and EGDe as well as their respective complement mutants. In contrast, 10403S *Δ0753* and EGDe *Δ0753* mutants showed an approximate 3-log reduction under the same treatment (*P*<0.0001). When treated with Tsunami 100, an approximate 2-log reduction was seen for both wild-type strains and their complements. Under the same treatment, 10403S *Δ0753* mutant showed an approximate 3.5-log reduction and EGDe *Δ0753* showed an approximate 4-log reduction (*P*<0.0001). These results collectively suggest that *lmo0753* plays a critical role in the surface attachment of *L. monocytogenes* on both lettuce leaves and cantaloupe rinds. In addition, Tsunami 100 appears to be more effective to inactivate *L. monocytogenes* attached on romaine lettuce and cantaloupe rind than chlorine.

### *L. monocytogenes* survival kinetics in soil

**Figure 4:**
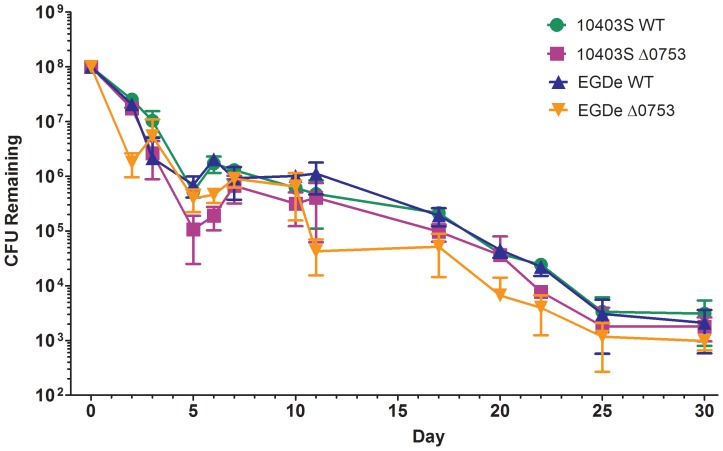
*L. monocytogenes* soil survival. Bacterial survival in potting soil at 25°C for 30 days. Standard deviations represent three independent experiments performed in triplicate.

To determine if *lmo0753* plays a role in the survival and persistence of *L. monocytogenes* in soil, we monitored the survival kinetics of 10403S *Δ0753* and EGDe *Δ0753* with their respective wild-type strains in potting soil over a 30-d period (Fig. 4). Generally, both 10403S and EGDe wild-types and their respective *Δ0753* mutants showed similar populations over 25 d in potting soil. For all strains, a decline of approximately 2-log was seen from day 1 to day 5, followed by a slight increase in 1-log from day 5 to day 7. A steady decline in approximately 2-log was seen from day 7 to day 25, and stabilizing from day 25 to day 30. The total population of 10403S *Δ0753* was approximately 1-log lower than its parent strain on days 5, 6, and 22 (*P*<0.0001). The total population of EGDe *Δ0753* was approximately 1-log lower than its parent strain on days 2, 11, and 20 (*P*<0.0001). Complementation mutants had no significant difference in their survival kinetics as compared with their wild-type phenotypes (data not shown).

## Discussion

In this study we demonstrated that *lmo0753* plays an important role in growth in *L. monocytogenes* strains10403S and EGDe, in particular, when grown with limited nutrients and at temperatures below 37°C. It has been shown that 10403S *ΔprfA* and prfA* display growth defects under some of the same conditions [17]. It has been demonstrated that prfA* mutants display a competitive defect when grown with its wild-type strain in nutrient-rich BHI broth [17].

Our findings also suggest that *lmo0753* contributes to biofilm formation in *L. monocytogenes*, which is an important mechanism for environmental persistence of this organism. Biofilm formation is a dynamic process and multiple factors contribute to the generation of biofilm by organisms. Sessile biofilm-associated cells are differentiated from their planktonic counterparts by the generation of an extracellular polymeric substance matrix with reduced growth rates and increased stress resistance to chemicals and disinfectants [18]–[21]. Bacterial biofilms have become a great public health concern because of their roles in food processing plants and the spread of pathogenic organisms to food products. It has been reported that certain serotypes, mainly 1/2a, 1/2b, and 4b, of *L. monocytogenes* are capable of persisting in RTE food processing plants for months or even years because of their advantageous biofilm properties [22], [23]. Common indigenous organisms present in food processing plant settings, such as *Staphylococcus capitis* and *Stenotrophomonas maltophilia*, can aid in the biofilm production by *L. monocytogenes* by approximately 1-log in binary systems [24]. Also, serotype 1/2a strains of *L. monocytogenes* is frequently implicated in contamination of RTE food products and human listeriosis cases [25], [26], likely due to its ability to form biofilms when nutrients are limited [27]. This study showed that deletion of *lmo0753* resulted in decreased biofilm formation on glass surface in nutrient-rich BHI medium. Interestingly, deletion of *prfA* from 10403S showed the same phenomenon as previously reported by Lemon *et al*. [15] and Zhou *et al*. [16]. We also discovered decreased biofilm formation by both *Δlmo0753* mutants on polystyrene surface in PW medium, collectively suggesting that *lmo0753* may play a major role in biofilm formation in *L. monocytogenes*.

Washing with sodium hypochlorite, or chlorine, is a common industrial practice to disinfect fresh produce surface. Chlorine is added to the wash water and acts as an oxidizer to help reduce the overall bacterial load and possible cross-contamination on minimally processed fruits and vegetables [28], [29]. These washing steps are considered to be important to ensure quality, safety, and shelf-life of the end products. Although chlorine has been a gold standard in industrial disinfectants, in recent years there have been trends to eliminate chlorine from the disinfection process because of concerns about efficacy and environmental risks [30], [31]. An alternative disinfectant agent for fresh produce wash waters is Ecolab Tsunami 100, the only EPA-registered antimicrobial water additive [32]. Tsunami 100 consists of acetic acid, peracetic acid, and hydrogen peroxide and is effective in eliminating *E. coli* O157:H7, *L. monocytogenes*, and *S. enterica* [32], [33]. The disinfectant also does not react with organic material, making it easier to use and maintain at a constant dosage in wash water compared to chlorine-based products. Using both sodium hypochlorite and Tsunami 100, we demonstrated that *lmo0753* plays important roles in both attachment to plant surfaces and in disinfectant resistance.

Previous studies have reported the survival rates of *L. monocytogenes* in various environmental niches such as water [34], [35], various surface materials [36]–[38], sewage and compost [39], [40], and soil [34], [41]–[44]. Soil is a major environmental reservoir for *L. monocytogenes* in which organic material ranges from 0.8 to 2.0% [45]. Piveteau *et al*. [44] showed that the gene expression profiles of *L. monocytogenes* altered drastically when inoculated into soil microcosms and soil extracts compared to culturing broth. Genes encoding transport and binding proteins, metabolism of amino acids, lipoproteins, and phage-related functions were up-regulated within 18 h of inoculation. This indicates that *L. monocytogenes* turns on a variety of transport and energy production mechanisms when environmental nutrients are limited. It has been reported that *L. monocytogenes* can persist in soil for months, with positive detection even after 1 year [44]. Our soil study showed that *L. monocytogenes* 10403S and EGDe and their respective *Δ0753* mutants were able to persist in potting soil for more than 30 days, with total populations decreased from 10^8^ to 10^4^ CFU/g. The ability of this pathogen to survive in soil for extended periods of time was not significantly influenced by the deletion of *lmo0753*.

## Materials and Methods

### Bacterial strains and culture conditions

All bacterial strains used in this study are described in Table 1. *L. monocytogenes* EGDe (ATCC# BAA-679) was obtained from the American Type Culture Collection (ATCC, Manassas, VA). 10403S, a Sm^r^ strain, was provided by the Nancy Frietag Laboratory. All other strains were derived from laboratory stocks. Strains derived from 10403S are Sm^r^. All strains were grown overnight at 37°C in Brain Heart Infusion (BHI) broth or Luria-Bertani (LB) broth (Becton, Dickinson and Co., Franklin Lakes, NJ) supplemented with streptomycin (200 µg ml^−1^) when necessary, prior to commencing experiments.

**Table 1:** Bacterial strains used in this study.

Strain	Designation	Reference
* L. monocytogenes* EGDe	Wild-type	
* L. monocytogenes* EGDe *Δ0753*		[49]
* L. monocytogenes* EGDe *Δ0753* (JS-c0753)		[49]
* L. monocytogenes* 10403S	Wild-type	
* L. monocytogenes* 10403S *Δ0753*		[49]
* L. monocytogenes* 10403S *Δ0753* (JS-c0753)		[49]
* L. monocytogenes* 10403S *ΔprfA*		[50]
* L. monocytogenes* 10403S prfA* (G145S)	Constitutively active mutant	[51], [52]

### Growth curves

Growth curves were performed in BHI, LB broth, and 1% peptone water (PW) (containing 3 g L^−1^ glucose). BHI broth was used as a nutrient-rich medium, LB was used as a nutrient-limiting medium, and PW was used as a minimum growth medium. These three media were used to compare the growth and maximum population levels of *L. monocytogenes* wild-type and mutant strains when subjected to different nutrient availabilities and incubation temperatures. Growth curves at incubation temperatures of 25°C or 37°C were performed using a Bioscreen C automatic growth curve system (Growth Curves, Piscataway, NJ). Growth curves at 4°C were performed manually. For Bioscreen C growth curves, bacterial growth was monitored by recording the cell turbidity every 5 min, after a 10 s shaking period, over a period of 48 h. For manual growth curves, the cell turbidity was recorded every two hours over a period of 48 h using a GENESYS 20 spectrophotometer (Thermo Fisher Scientific, Inc., Waltham, MA). Experiments were performed at least three times with quadruplicate samples and verified by plate counting on Brilliance *Listeria* Agar (Remel Inc., Lenexa, KS) at time points 0, 4, 8, 16, 24, 32, and 48 h.

### Biofilm production assays

Biofilm assays were assessed using the crystal violet assay method as previously described [15], [16], [46], [47] with modifications. Assays were performed using BHI nutrient-rich medium and PW minimum growth medium to compare biofilm production when subjected to varying nutrient availabilities. Briefly, 48 h cultures in BHI or PW in glass test tubes or a 96-well polystyrene microtiter plate were washed three times with PBS and stained for 15 min with a 1% aqueous crystal violet solution. Test tubes and wells were washed again three times with PBS and then incubated with 95% ethanol for 20 min. OD_570_ readings, which reflect the amount of biofilm formed by the attached bacteria, were measured. The experiment was performed at least three times with triplicate samples for statistical analysis.

### Attachment assays to romaine lettuce and cantaloupe rind

Attachment assays were conducted for each wild-type strain and its corresponding deletion and complementation mutant, as previously described [47], [48] with minor modifications. Romaine lettuce and cantaloupe were purchased from a local retail grocer, stored at 4°C, and used within 48 h. *L. monocytogenes* cultures were grown overnight in BHI at 37°C with shaking. All cultures were normalized to 1×10^8^ CFU/ml and 1 ml was added into a 50-ml conical tube containing 45 ml PBS. For each experiment, nine pieces of romaine lettuce leaves (approximately 1 g each) were placed into the conical tubes and incubated at room temperature with *L. monocytogenes* culture for 10 min. After incubation, leaves were pulled out and air dried at room temperature in a biohazard cabinet in sterile petri dishes for 1 h. Three leaves were washed three times with PBS as the untreated control; three leaves were immersed in a 100 ppm aqueous chlorine solution (made from a 13% sodium hypochlorite stock solution) for 10 s and the reaction was stopped by adding 4.5 ml of 1 M sodium thiosulfate; and three leaves were immersed in a 100 ppm aqueous Tsunami 100 (Ecolab, St. Paul, Minnesota) solution for 10 s and the reaction was stopped by the addition of 10 ml of Dey/Engley Neutralizing Broth (Remel Inc., Lenexa, KS). The chlorine- and Tsunami-treated leaves were then washed three times with PBS. To recover attached bacteria, five sterile 6-mm glass beads were added to the leaves in 50-ml conical tubes containing 10 ml PBS and vortexed vigorously for 1 min. Serial dilutions of the eluted bacteria were plated on BHI agar (Becton Dicksinson and Co.) and Brilliance *Listeria* Agar (Remel Inc., Lenexa, KS) in duplicate. The plates were incubated at 37°C for 24 or 48 h before CFU were enumerated. Attachment assays with cantaloupe rind were performed similarly using nine 1 in.×1 in. pieces of rind (approximately 5 g each) for each experiment. Instead of dip inoculation, 1 ml of culture was spot inoculated on the outside of cantaloupe rind and allowed to air dry for 1 h as previously described. All attachment experiments were conducted independently at least three times in triplicate for statistical analysis.

### Soil survival assays

Potting soil was purchased from a local retail garden center and stored at room temperature. Soil assays were conducted similarly to that previously described [41] with modifications. 1 g of soil was placed into 20-ml glass test tubes and autoclaved to ensure sterility. *L. monocytogenes* cultures were grown overnight in BHI at 37°C with shaking. All cultures were normalized to 1×10^8^ CFU/ml and 1 ml was added into each test tube. At time points ranging from 2 to 5 d, test tubes in triplicate for each wild-type, mutant, and complement, were suspended in 10 ml PBS along with five 6-mm sterile glass beads and vortexed vigorously for one min. Serial dilutions of the eluted bacteria were plated on BHI agar and Brilliance *Listeria* Agar in duplicate. The plates were incubated at 37°C for 24 or 48 h before CFU were enumerated. All experiments were performed independently three times in triplicate for statistical analysis.

### Statistical analysis

Student's *t*-test and ANOVA analysis were performed using GraphPad Prism software package (GraphPad Software, Version 5) and Excel software (Microsoft, Version 2010). A *P*-value less than 0.05 was considered a significant difference.

## Supporting Information

Figure S1 **Standard growth curve at 37°C of** ***Listeria monocytogenes*** **10403S wild-type strain with linear regression.** Standard curves were performed three times with triplicate samples. Averages of triplicates for each experiment are shown.
(TIF)
