# Supplementary figures and images for: Roles of a Novel Crp/Fnr Family Transcription Factor Lmo0753 in Soil Survival, Biofilm Production and Surface Attachment to Fresh Produce of Listeria monocytogenes
Source: PLoS One. 2013 Sep 16;8(9):e75736. doi: 10.1371/journal.pone.0075736 (PMC3774658; doi:10.1371/journal.pone.0075736)

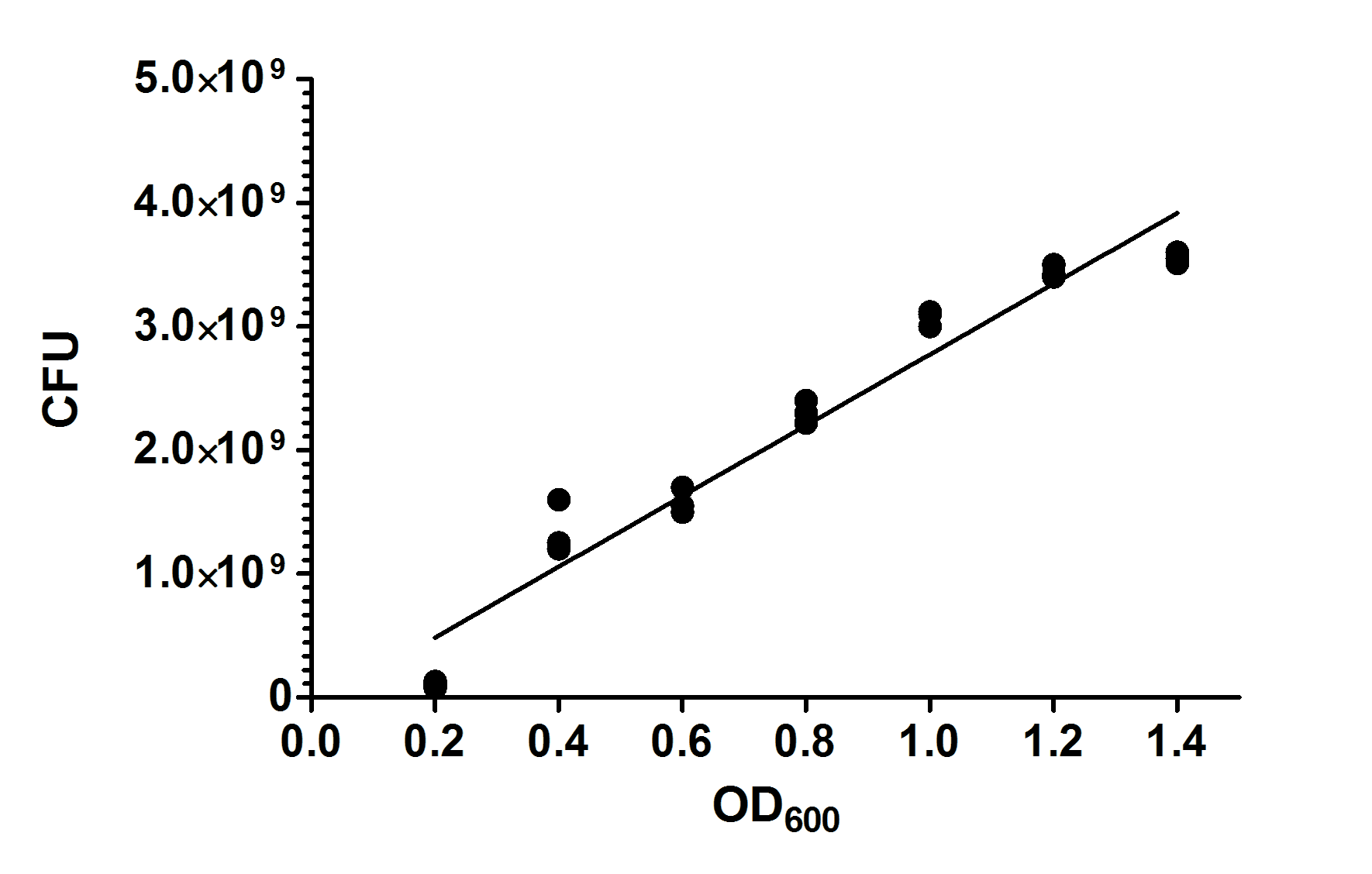

Supplement: Figure S1 — Standard growth curve at 37°C of Listeria monocytogenes 10403S wild-type strain with linear regression. Standard curves were performed three times with triplicate samples. Averages of triplicates for each experiment are shown. (TIF) [file pone.0075736.s001.tif]
